# Medical Institute of Louisville

**Published:** 1845-11

**Authors:** 


MEDICAL INSTITUTE OF LOUISVILLE.

The lectures in this institution will commence with an introductory address by Professor Short, on Monday, the 3d instant. More students have arrived in the city, and entered their names on the matriculation book of the school than were ever before here by the beginning of November, which would seem to promise that there will be an increase upon the large class of last winter. This, we believe, is more than the friends of the school expected; it was doubted whether the growth of last year ought not to be regarded as the growth of two years. We think it will not turn out so, but that the increase, this season, will be nearly if not quite equal to that of the last. Y

